# Supplementary material for: Genome-Wide Analysis of the LATERAL ORGAN BOUNDARIES Domain (LBD) Members in Alfalfa and the Involvement of MsLBD48 in Nitrogen Assimilation
Source: Int J Mol Sci. 2023 Feb 28;24(5):4644. doi: 10.3390/ijms24054644 (PMC10003661; doi:10.3390/ijms24054644)
Supplement: Supplementary file 1 [file ijms-24-04644-s001.zip › ijms-2121867-supplementary.pdf]

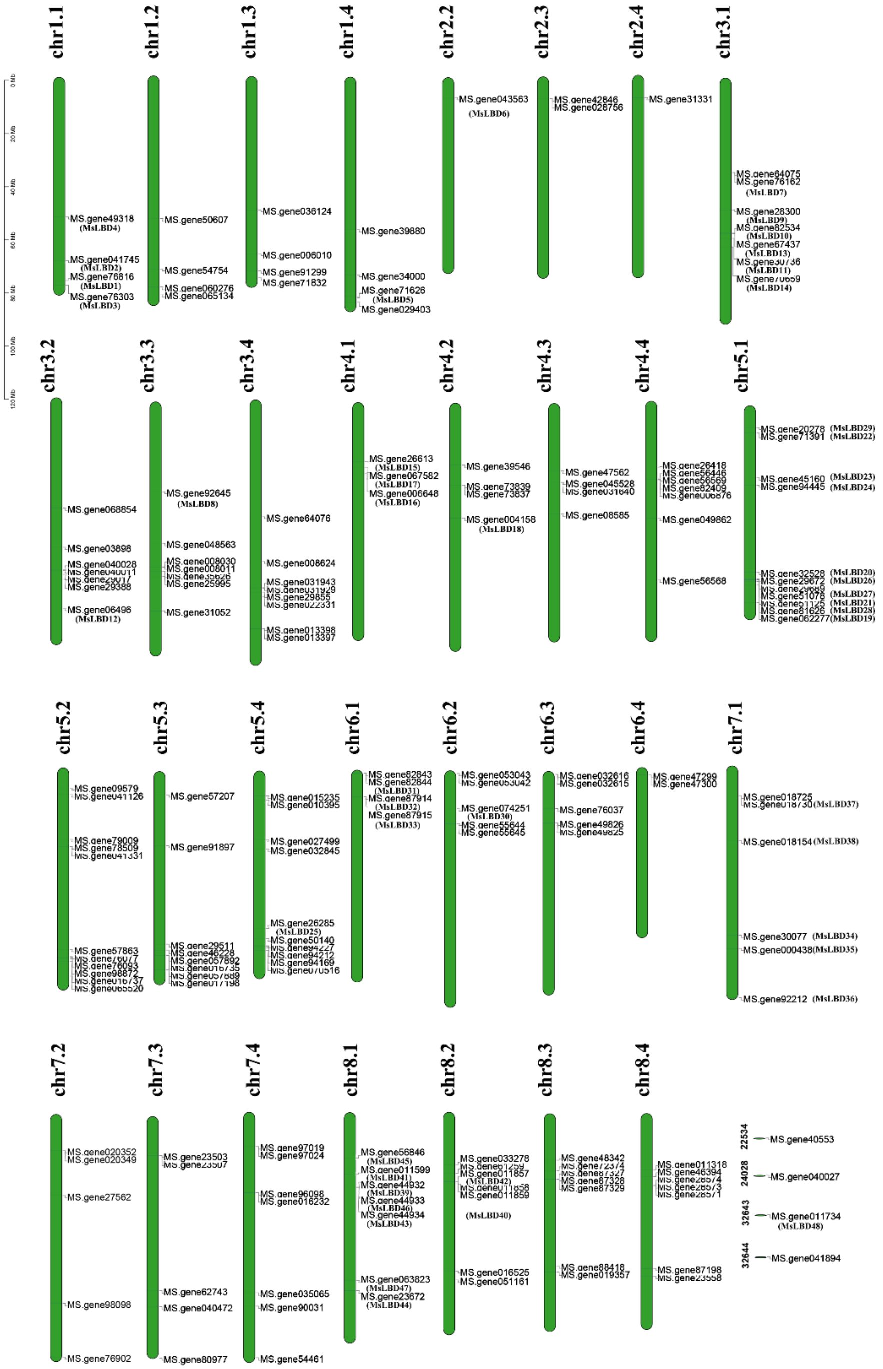

Figure S1. Distribution of the identified *MsLBDs* in alfalfa chromosome. MsLBDs, alfalfa lateral organ boundaries domain genes.

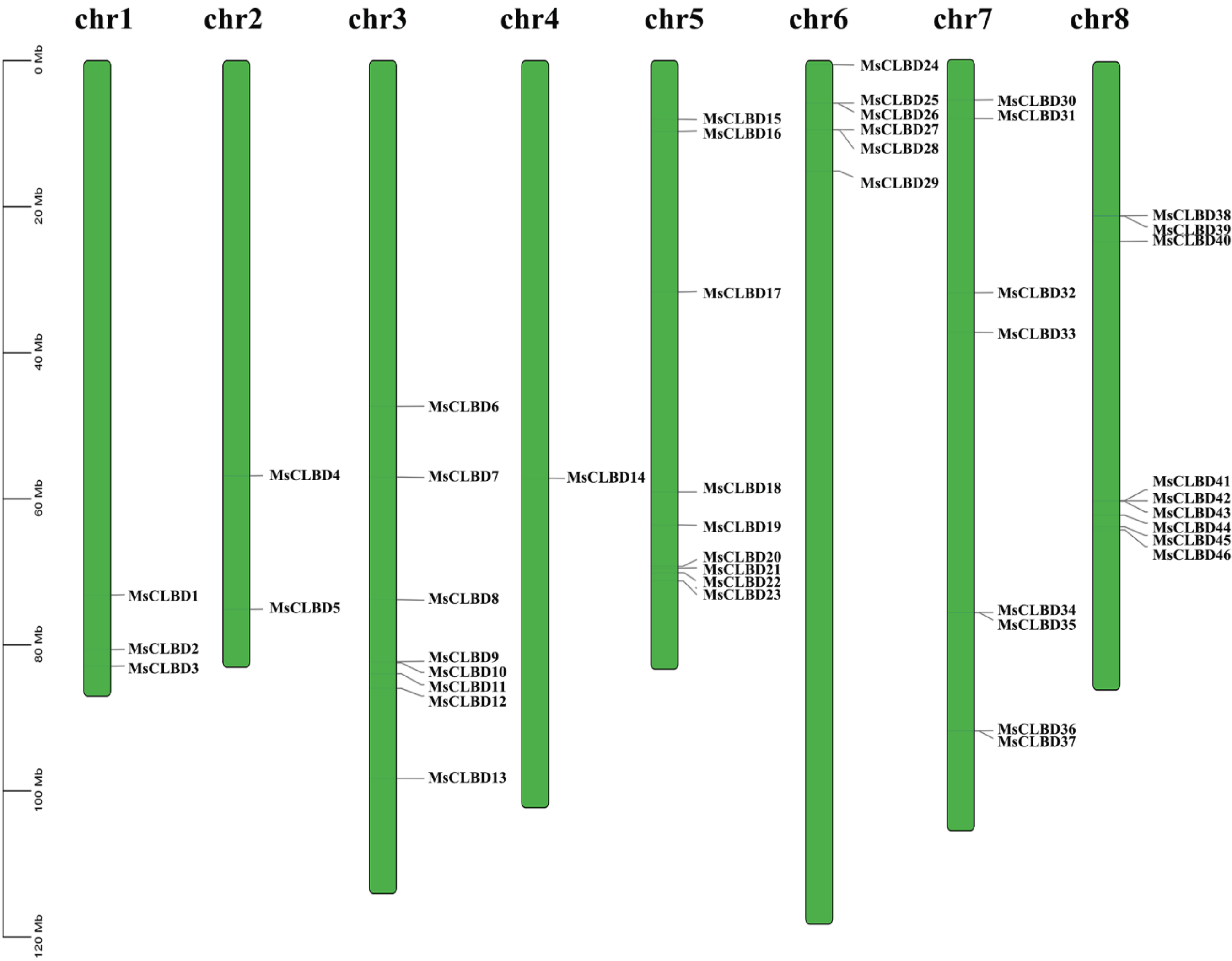

Figure S2. Distribution of the *MsCLBD*s in *M. sativa spp. Caerulea* chromosome. MsCLBDs, *M. sativa spp. Caerulea* lateral organ boundaries domain genes.

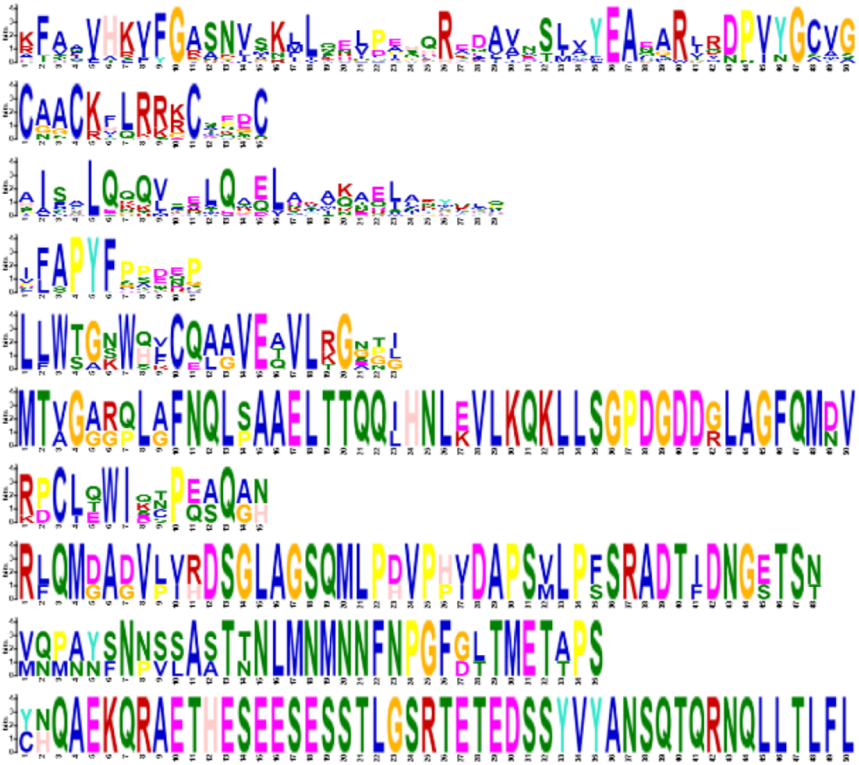

	Logo	E-value	Sites	Width
1.		1.8e-1265	45	50
2.		1.2e-439	48	15
3.		1.9e-240	35	29
4.		4.7e-193	41	11
5.		3.9e-059	7	23
6.		7.1e-039	3	50
7.		9.4e-033	7	15
8.		1.0e-032	3	48
9.		2.3e-021	3	35
10.		2.7e-013	2	50

Figure S3. Conserved motifs of MsLBDs proteins

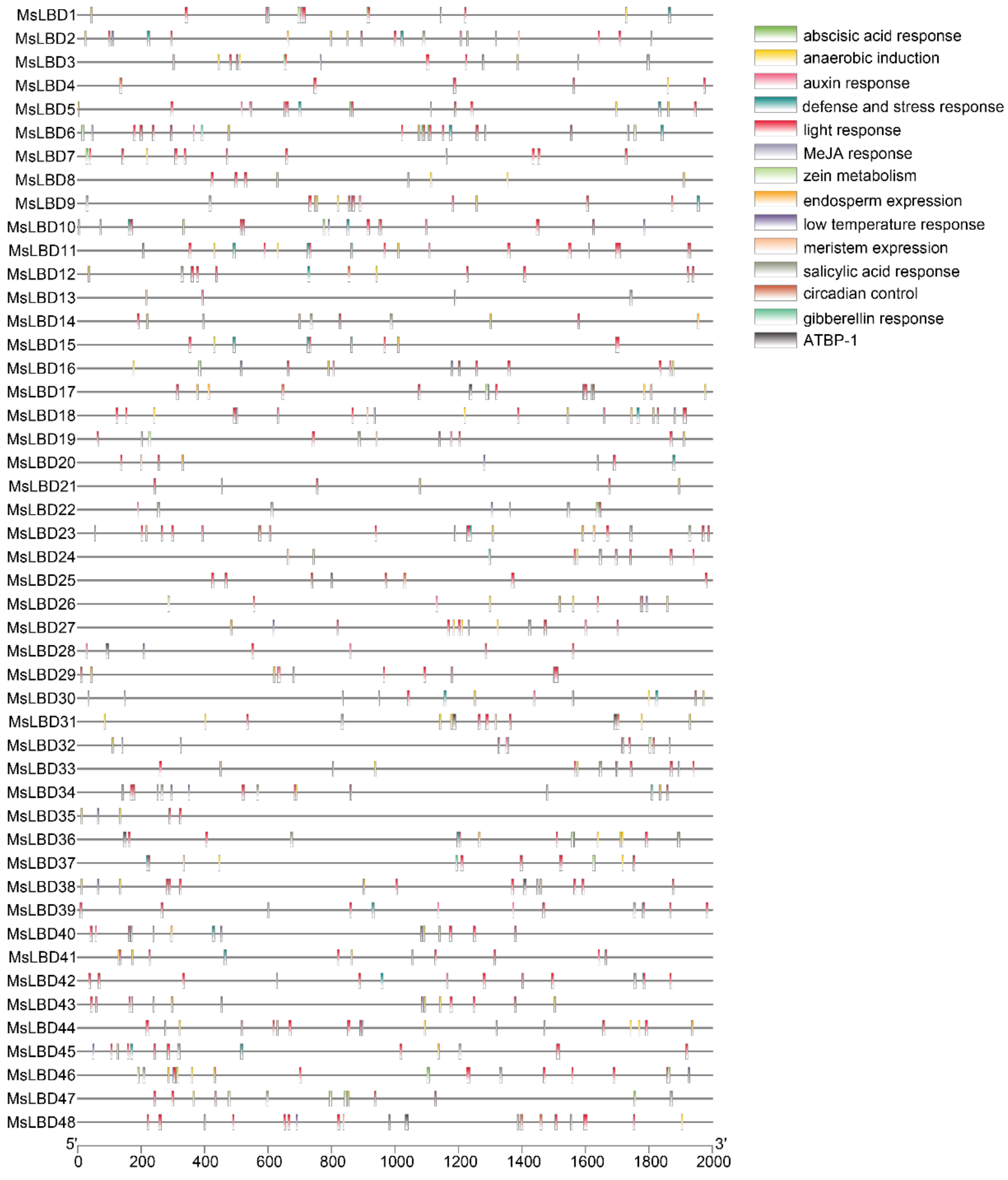

Figure S4. Promoter element of *MsLBD*s genes

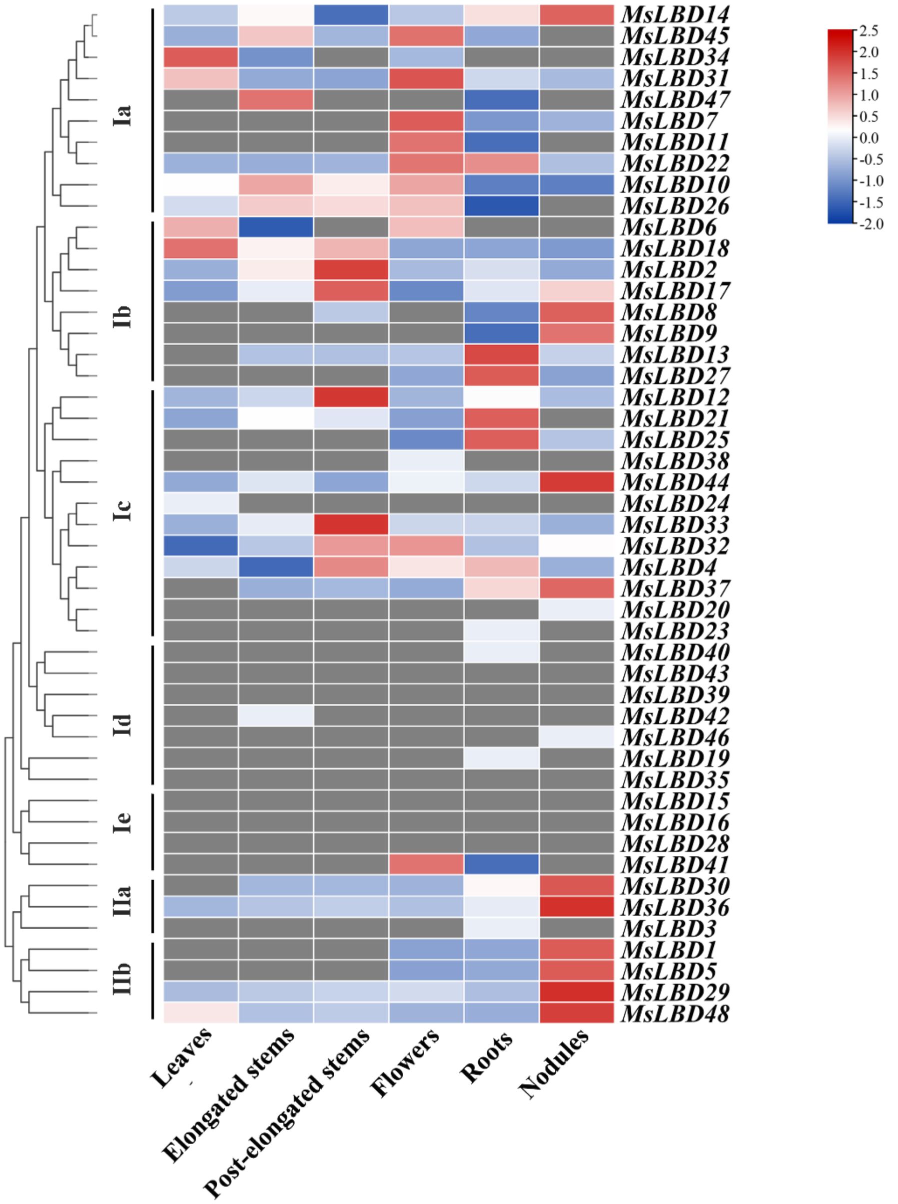

Figure S5. Heatmap of the expression profile of *MsLBD* genes. The relative expression of each *MsLBD* gene was normalized by TBtools software in different tissue. Blue indicated down-regulation, red for up-regulation, the grey for genes undetected in the RNA sequencing data.

Table S1 Basic information of the putative MsLBDs in alfalfa

Gene_name	Gene_id	Location	Size	MW(kDa)	pI	Location prediction
MsLBD14	MS.gene022331	chr3.4:74172606:74173187	193 a.a.	20.94	7.63	nucleus
MsLBD45	MS.gene011318	chr8.4:21137334:21137906	190 a.a.	20.71	7.63	nucleus
MsLBD34	MS.gene30077	chr7.1:63714330:63714848	172 a.a.	18.77	5.52	nucleus
MsLBD31	MS.gene032616	chr6.3:1161920:1162375	151 a.a.	16.62	6.57	nucleus
MsLBD47	MS.gene87198	chr8.4:58297179:58297874	231 a.a.	25.39	8.26	nucleus
MsLBD7	MS.gene068854	chr3.2:41474825:41475718	297 a.a.	31.9	5.91	nucleus
MsLBD11	MS.gene35626	chr3.3:63690097:63690793	204 a.a.	22.72	8.71	nucleus
MsLBD22	MS.gene010395	chr5.4:10916826:10917782	318 a.a.	34.77	6.56	nucleus
MsLBD10	MS.gene008030	chr3.3:62078169:62078726	185 a.a.	20.59	8.1	nucleus
MsLBD26	MS.gene94227	chr5.4:65755991:65756530	179 a.a.	19.98	6.81	nucleus
MsLBD6	MS.gene31331	chr2.4:8424586:8427204:-	174 a.a.	19.46	5.16	nucleus
MsLBD18	MS.gene049862	chr4.4:44081649:44083790	185 a.a.	20.26	5.49	nucleus
MsLBD2	MS.gene54754	chr1.2:72649812:72651370	230 a.a.	25.22	6.43	nucleus
MsLBD17	MS.gene56569	chr4.4:29258656:29262353	174 a.a.	18.92	7.7	nucleus
MsLBD8	MS.gene92645	chr3.3:33819966:33823100	147 a.a.	16.56	8.54	nucleus
MsLBD9	MS.gene048563	chr3.3:53189343:53190643	176 a.a.	19.7	5.88	nucleus
MsLBD13	MS.gene031929	chr3.4:70837285:70838290	160 a.a.	18.06	7.64	nucleus
MsLBD27	MS.gene29689	chr5.1:65545351:65546630	166 a.a.	18.69	6.5	nucleus
MsLBD12	MS.gene31052	chr3.3:78615404:78616107	205 a.a.	22.45	8.63	nucleus
MsLBD21	MS.gene98872	chr5.2:71826185:71827588	196 a.a.	21.83	7.64	nucleus
MsLBD25	MS.gene26285	chr5.4:59341388:59342282	142 a.a.	15.56	6.41	nucleus
MsLBD38	MS.gene96098	chr7.4:30185115:30188016	197 a.a.	21.91	6.58	nucleus
MsLBD44	MS.gene019357	chr8.3:59598280:59606588	259 a.a.	28.31	6.49	nucleus
MsLBD24	MS.gene032845	chr5.4:29480141:29480862	202 a.a.	21.58	9.35	nucleus
MsLBD33	MS.gene47300	chr6.4:2862808:2863768	227 a.a.	24.44	9.2	nucleus
MsLBD32	MS.gene49826	chr6.3:19474848:19475838	209 a.a.	23.06	8.78	nucleus
MsLBD4	MS.gene39880	chr1.3:50204038:50205951	204 a.a.	22.38	6.79	nucleus
MsLBD37	MS.gene23507	chr7.3:14703507:14704706	218 a.a.	24.15	7.05	nucleus
MsLBD20	MS.gene57863	chr5.2:68313632:68314864	194 a.a.	21.3	5.67	nucleus
MsLBD23	MS.gene027499	chr5.4:26000842:26002006	224 a.a.	25.16	5.94	nucleus
MsLBD40	MS.gene011859	chr8.2:26259985:26260641	218 a.a.	24.31	5.76	nucleus
MsLBD43	MS.gene87329	chr8.3:24663882:24664565	227 a.a.	25.15	5.57	nucleus
MsLBD39	MS.gene44932	chr8.1:28095019:28095934	231 a.a.	24.87	5.05	nucleus
MsLBD42	MS.gene87327	chr8.3:24626480:24627396	271 a.a.	29.32	5.5	nucleus
MsLBD46	MS.gene28573	chr8.4:27097987:27098778	263 a.a.	28.09	6.24	nucleus
MsLBD19	MS.gene062277	chr5.1:74140421:74141954	157 a.a.	18.1	8.98	nucleus
MsLBD35	MS.gene98098	chr7.2:71052146:71052981	157 a.a.	18.01	8.23	nucleus
MsLBD15	MS.gene39546	chr4.2:23249084:23249914	276 a.a.	31.48	8.74	nucleus
MsLBD16	MS.gene045528	chr4.3:29689620:29690594	196 a.a.	22.78	8.75	nucleus
MsLBD28	MS.gene057889	chr5.3:69179502:69180203	233 a.a.	26.31	5.1	nucleus
MsLBD41	MS.gene72374	chr8.3:21407143:21407616	157 a.a.	17.37	7.65	nucleus
MsLBD30	MS.gene074251	chr6.2:14344708:14346344	278 a.a.	30.54	8.03	nucleus
MsLBD36	MS.gene76902	chr7.2:91305148:91306331	301 a.a.	32.99	8.07	nucleus
MsLBD3	MS.gene71832	chr1.3:75557737:75558748	231 a.a.	25.24	8.99	nucleus
MsLBD1	MS.gene76816	chr1.1:76542635:76543444	212 a.a.	23.8	5.74	nucleus
MsLBD5	MS.gene71626	chr1.4:82355009:82355729	179 a.a.	19.96	5.93	nucleus
MsLBD29	MS.gene57207	chr5.3:8766360:8767197	231 a.a.	25.2	8.62	nucleus
MsLBD48	MS.gene041894	32644:18066:18887	233 a.a.	25.29	8.1	nucleus

Note: *M. sativa LATERAL ORGAN BOUNDARIES DOMAIN transcription factors*(MsLBDs).

Table S2 Preliminary information of the putative MsCLBDs in *M. sativa spp. Caerulea*

Gene name	Gene_id	Location	Size	MW（kDa）	pI	Location prediction
MsCLBD12	MsaT015364	Chr3：85959009：85959590	193 a.a.	20.93	7.8	nucleus
MsCLBD26	MsaT030281	Chr6：5819528：5819983	151 a.a.	16.62	6.57	nucleus
MsCLBD27	MsaT030282	Chr6：5831713：5832141	142 a.a.	15.84	6.18	nucleus
MsCLBD31	MsaT036548	Chr7：37357894：37358412	172 a.a.	18.83	5.52	nucleus
MsCLBD40	MsaT042594	Chr8：24599747：24600418	223 a.a.	24.47	8.26	nucleus
MsCLBD7	MsaT013595	Chr3：57010098：57010985	295 a.a.	31.78	5.83	nucleus
MsCLBD11	MsaT015211	Chr3：83940752：83941358	204 a.a.	22.75	8.71	nucleus
MsCLBD23	MsaT025540	Chr5：9684729：9685685	318 a.a.	34.77	6.56	nucleus
MsCLBD18	MsaT028821	Chr5：69255786：69256319	177 a.a.	19.79	6.81	nucleus
MsCLBD9	MsaT015066	Chr3：82291736：82292293	185 a.a.	20.59	8.1	nucleus
MsCLBD8	MsaT014481	Chr3：73792224：73792595	176 a.a.	19.69	5.88	nucleus
MsCLBD10	MsaT015081	Chr3：82450564：82450881	159 a.a.	17.92	7.64	nucleus
MsCLBD19	MsaT028836	Chr5：69470495：69470839	166 a.a.	18.69	6.5	nucleus
MsCLBD6	MsaT013281	Chr3：47321941：47322096	145 a.a.	16.3	7.61	nucleus
MsCLBD4	MsaT009292	Chr2：56843376：56843534	164 a.a.	18.36	8.28	nucleus
MsCLBD1	MsaT004729	Chr1：73159722：73160078	230 a.a.	25.22	6.43	nucleus
MsCLBD5	MsaT010576	Chr2：75127349：75127708	198 a.a.	22.1	5.2	nucleus
MsCLBD14	MsaT020688	Chr4：57175175：57175369	185 a.a.	20.26	5.49	nucleus
MsCLBD13	MsaT016470	Chr3：98270810：98271001	122 a.a.	13.51	8.61	nucleus
MsCLBD20	MsaT028887	Chr5：70118385：70118398	196 a.a.	21.8	7.64	nucleus
MsCLBD17	MsaT028528	Chr5：63567376：63567401	180 a.a.	19.73	8.11	nucleus
MsCLBD38	MsaT042385	Chr8：21091421：21091448	273 a.a.	29.36	8.76	nucleus
MsCLBD34	MsaT038370	Chr7：75720732：75721124	236 a.a.	24.74	8.72	nucleus
MsCLBD39	MsaT042387	Chr8：21145858：21146196	216 a.a.	23.6	7.1	nucleus
MsCLBD33	MsaT038369	Chr7：75694211：75694510	195 a.a.	21.61	6.58	nucleus
MsCLBD37	MsaT039690	Chr7：91946121：91946402	223 a.a.	24.93	5.93	nucleus
MsCLBD36	MsaT039686	Chr7：91909434：91909790	218 a.a.	24.14	7.05	nucleus
MsCLBD16	MsaT028214	Chr5：59062539：59062817	193 a.a.	21.26	5.84	nucleus
MsCLBD29	MsaT030527	Chr6：9459093：9459443	209 a.a.	23.06	8.78	nucleus
MsCLBD28	MsaT030526	Chr6：9454875：9455180	227 a.a.	24.44	9.2	nucleus
MsCLBD15	MsaT026939	Chr5：31658872：31659246	202 a.a.	21.6	9.35	nucleus
MsCLBD30	MsaT036316	Chr7：31931914：31932234	156 a.a.	17.82	8.23	nucleus
MsCLBD25	MsaT029978	Chr6：570228：570248	241 a.a.	27.22	7.55	nucleus
MsCLBD21	MsaT028964	Chr5：71237317：71238012	231 a.a.	26.25	5.68	nucleus
MsCLBD46	MsaT044729	Chr8：64091129：64091599	156 a.a.	17.26	8.21	nucleus
MsCLBD42	MsaT044430	Chr8：60119992：60120492	166 a.a.	18.88	6.9	nucleus
MsCLBD45	MsaT044701	Chr8：63676605：63676646	86 a.a.	9.65	7.73	nucleus
MsCLBD41	MsaT044429	Chr8：60093732：60094049	220 a.a.	23.61	6.58	nucleus
MsCLBD43	MsaT044434	Chr8：60184159：60184953	264 a.a.	28.91	5.6	nucleus
MsCLBD44	MsaT044566	Chr8：62087191：62087374	233 a.a.	25.32	7.53	nucleus
MsCLBD22	MsaT025414	Chr5：8043179：8043690	231 a.a.	25.07	8.4	nucleus
MsCLBD2	MsaT005365	Chr1：80640560：80641011	211 a.a.	23.66	5.96	nucleus
MsCLBD3	MsaT005565	Chr1：82906029：82906528	231 a.a.	25.24	8.99	nucleus
MsCLBD24	MsaT030850	Chr6：15116464：15117110	278 a.a.	30.43	8.03	nucleus
MsCLBD35	MsaT035149	Chr7：8072569：8072758	301 a.a.	32.99	8.56	nucleus
MsCLBD32	MsaT034983	Chr7：5538972：5539161	301 a.a.	32.96	8.07	nucleus

Note: *M. sativa spp. Caerulea LATERAL ORGAN BOUNDARIES DOMAIN transcription factors*(MsCLBDs).

Table S3 Classification of the LBD members in select species.

	M. sativa L. Xinjiandaye (4x = 32)	*M. sativa spp. Caerulea* (2x = 16)	*M. truncatula* (2x = 16)	*A.thaliana* (2x = 10)	Average
Genome (Mb)	3,150	793.2	420	125	
No. LBDs	48	46	57	42	48.25
**Class I**					
I a	10 (20.83%)	10 (21.74%)	11 (19.30%)	6 (14.29%)	19.04%
I b	11 (22.92%)	11 (23.91%)	11 (19.30%)	7 (16.67%)	20.70%
I c	9 (18.75%)	10 (21.74%)	11 (19.30%)	10 (23.81%)	20.90%
I d	7 (14.58%)	5 (10.87%)	5 (8.77%)	5 (11.90%)	11.53%
I e	4 (8.33%)	3 (6.52%)	10 (17.54%)	8 (19.05%)	12.86%
sum	41 (85.42%)	39 (84.78%)	48 (84.21%)	36 (85.71%)	85.03%
**Class II**					
II a	3 (6.25%)	4 (8.70%)	6 (10.53%)	3 (7.14%)	8.15%
II b	4 (8.33%)	3 (6.52%)	3 (5.26%)	3 (7.14%)	6.82%
sum	7 (14.58%)	7 (15.22%)	9 (15.79%)	6 (14.29%)	14.97%

Table S4 Homology analysis of MsLBDs from the different subclasses.

		I a	I b	I c	I d	I e	II a	II b	
	maximum / minimum	98.80%	79%	74.10%	64.20%	50.60%	30.90%	32.10%	I a
I a	60.50%		91.40%	79%	61.70%	48.10%	28.40%	30.90%	I b
I b	58%	65.40%		95.10%	59.30%	45.70%	29.60%	30.90%	I c
I c	48.10%	43.20%	48.10%		98.80%	44.40%	27.20%	27.20%	I d
I d	46.90%	45.70%	39.50%	42%		70.40%	29.60%	33.30%	I e
I e	33.30%	35.80%	28.40%	30.90%	30.90%		91.40%	72.80%	II a
II a	25.60%	22.20%	23.50%	21%	18.50%	66.70%		98.80%	II b
II b	27.20%	25.90%	21%	21%	21%	54.30%	76.50%		
	I a	I b	I c	I d	I e	II a	II b		

Table S5 Homology analysis of MsCLBDs from the different subclasses.

		I a	I b	I c	I d	I e	II a	II b	
	maximum<br>minimum	98.00%	73.50%	74.10%	59.20%	61.20%	32.70%	26.50%	I a
I a	55.10%		89.80%	77.60%	57.10%	57.10%	30.60%	26.50%	Ib
I b	53.10%	65.30%		93.90%	57.10%	53.10%	38.80%	28.60%	Ic
Ic	46.90%	42.90%	46.90%		77.6%	55.10%	24.50%	20.40%	Id
Id	40.80%	40.80%	32.70%	42.90%		79.60%	26.50%	20.40%	Ie
Ie	36.70%	38.80%	34.70%	36.70%	53.10%		95.90%	73.50%	II a
IIa	20.40%	22.40%	22.40%	18.40%	18.40%	75.50%		98.67%	II b
IIb	16.30%	16.30%	18.40%	16.30%	14.30%	51.00%	67.30%		
	I a	I b	I c	I d	I e	II a	II b		

Table S6 sequence homology of MsLBD48 with three Arabidopsis LBD homologues

protein	identity			
MsLBD48	100%			
AtLBD37	61.60%	100%		
AtLBD38	63.00%	83.10%	100%	
AtLBD39	63.50%	63.00%	64.80%	100%

Table S7 sequence of primers used in this study

Gene	Purpose/details	Primer sequence (5’ to 3’)
MsLBD1	qRT-PCR	F: 5’-GCAGTTGGGCTGTTATGGA-3’<br>R: 5’-ATCAGGGAGTGCGGTCAAT-3’
MsLBD3	qRT-PCR	F: 5’-TGAAGCCCAAGCCACGAGTT-3’<br>R: 5’-AAGCCAAGAGTGAGTTCCAAAT-3’
MsLBD5	qRT-PCR	F: 5’-TAACGCCACTGTCTTTGTC-3’<br>R:5’-CCCAACTGCTCCATTTACT-3’
MsLBD18	qRT-PCR	F: 5’-CTTCCCATAGCAATGGACAT-3’<br>R: 5’-TGAGCATAGGCTAGTGTTGT-3’
MsLBD29	qRT-PCR	F: 5’-GCGGCGACGACTGATCTTA-3’<br>R:5’-TCCTCCCATCCCACTTTCC-3’
MsLBD30	qRT-PCR	F: 5’-AAGGGTGTAGTGAAGATTGTAAC-3’<br>R:5’-GATGAGACCAGCACGACCA-3’
MsLBD36	qRT-PCR	F: 5’-AAAGCCCTGAATCACAAGC-3’<br>R:5’-ACCAGCACGACCGTAGAAT-3’
MsLBD48	qRT-PCR	F: 5’-TGGCGCTGCTGCTATTTGG -3’<br>R:5’-CGTTCCTCCACTGCCTTCTGT-3’
AtNRT1.1	qRT-PCR	F: 5’-CTGCCACACACTGAACAATTCC-3’<br>R:5’-CCCGCTTCCTGATCCCTTAT-3’
AtNRT2.1	qRT-PCR	F: 5’-TTGCTTTCTCGGTGCAATCAC-3’<br>R:5’-TGCTCTGTGTCCACCGGAA -3’
AtNIA1	qRT-PCR	F: 5’-GGCTACGCTTATTCTGGAGGAGGT-3’<br>R: 5’-TGGTGGTCAAGCTCACAAACACTC-3’
AtNIA2	qRT-PCR	F: 5’-GCCGACGAAGAAGGTTGGTGGTAT-3’<br>R: 5’-GAAGAATCTCCTCGTGACATGGCG-3’
MsLBD48	Isolation of CDS	F: 5’-AACAATGATGTCGTCGTGTAAT-3’<br>R:5’-TCATCATATAAGATTACCCTCTCCA-3’
MsLBD48	over expression	F: 5’-gaacacgggggactcttgacATGGACAAAGCATGGGGT-3’<br>R: 5’-atttaccctcagatctaccatGTTTCCTGAAAAGTTGCT-3’
MsLBD48	GFP	F: 5’-agaacacgggggactcttgaATGGACAAAGCATGGGGT-3’<br>R: 5’-cctttactagtcagatctaccatGTTTCCTGAAAAGTTGCT-3’
